# The Insurance of Nurses

**Published:** 1912-06-15

**Authors:** T. H. F. Lapthorn

**Affiliations:** Chairman of Committee of Management. The Royal Portsmouth Hospital, Portsmouth


*To the Editor of* The Hospital.

### The Insurance of Nurses.

### A HOSPITAL CHAIRMAN'S SUGGESTIONS.

*To the Editor of* The Hospital.

Sir,—Although this letter was written before I saw the articles on "The Insurance of Hospital Nurses" in your recent issues, I still venture to ask you to insert it, because I think the question whether nurses should at once proceed to join an "approved" society is well worth further discussion. It is a matter of vital interest to them how they can avoid paying under the Act for medical attendance which they at present, in practice, get for nothing.

You have already explained the effect of Section 15 (3) and (4) of the Act, but, with your permission, I will recapitulate their provisions. By the former all insured persons may, with the consent of the Insurance Committee, make their own arrangements for medical attendance, instead of receiving it through the Committee; and, if they do this, the Committee is bound to pay the cost if it does not exceed what it would have cost the Committee themselves to provide the medical attendance. And by the latter sub-section, in the case of nurses entitled to receive medical attendance through any approved institution now in existence, such medical attendance may be treated as, or as part of, their medical benefit under the Act, and the Insurance Committee may contribute towards the cost.

Under one of these sub-sections I think it should be quite possible to recover the cost of medical attendance on nurses from the Insurance Committee.

Let it be a part of each nurse's contract that the hospital shall supply her with medical attendance at a charge, say, of 6d. a month—then apply to the Insurance Committee to refund this sum as the cost of her medical attendance.

The Hospital Committee could then afford to raise each nurse's salary by the same amount, namely, 6d. a month, without being a penny out of pocket, because this increase in salary would be offset by the contribution from the Insurance Committee.

In this way the nurse's real payment under the Act would be only 7s., instead of 13s., a year.

I think nurses will be unwise to be in a hurry to join an "approved" society; and if they do so at once their contributions would necessarily be 13s. a year; while, if the plan which I have suggested proves workable, they ought to be able to get all the benefits of the Act for less than this.

The two societies being promoted in the nursing profession might well consider the question of offering reduced terms for the remaining benefits under the Act to those nurses who are able, in some such way as I have suggested above, to make their own arrangements for medical attendance.—Yours faithfully,

T. H. F. Lapthorn,
Chairman of Committee of Management.

The Royal Portsmouth Hospital, Portsmouth,
June 5.

